# Environmental Enrichment and Agonistic Behavior in Post-Weaning Pigs: A Pilot Study Using Artificial Intelligence

**DOI:** 10.3390/biology15030255

**Published:** 2026-01-30

**Authors:** Md Kamrul Hasan, Hong-Seok Mun, Keiven Mark B. Ampode, Eddiemar B. Lagua, Md Sharifuzzaman, Jin-Gu Kang, Young-Hwa Kim, Ahsan Mehtab, Hae-Rang Park, Chul-Ju Yang

**Affiliations:** 1 Animal Nutrition and Feed Science Laboratory, Department of Animal Science and Technology, Sunchon National University, Suncheon 57922, Republic of Korea; kamrul.ps@sau.ac.bd (M.K.H.); eddiemarlagua@gmail.com (E.B.L.); baushossain@gmail.com (M.S.);; 2 Department of Poultry Science, Sylhet Agricultural University, Sylhet 3100, Bangladesh; 3 Department of Multimedia Engineering, Sunchon National University, Suncheon 57922, Republic of Korea; 4 College of Agriculture, Forestry, and Environmental Sciences, Western Philippines University, Aborlan 5302, Palawan, Philippines; 5 Interdisciplinary Program in IT-Bio Convergence System (BK21 Plus), Sunchon National University, Suncheon 57922, Republic of Korea; 6 Department of Animal Science and Veterinary Medicine, Gopalganj Science and Technology University, Gopalganj 8105, Bangladesh; 7 Interdisciplinary Program in IT-Bio Convergence System (BK21 Plus), Chonnam National University, Gwangju 61186, Republic of Korea; 8 School Education Department, Narowal 51600, Punjab, Pakistan

**Keywords:** environmental enrichment, agonistic behavior, pig, AI monitoring, welfare

## Abstract

Weaning is a major stressor for pigs, often increasing agonistic behaviors such as aggression, ear biting, and tail biting, which can impair growth and welfare. This study evaluated the combined effect of rubber sticks and Italian ryegrass hay as environmental enrichment (EE) on growth performance, agonistic behavior, ear and tail biting lesion development, fecal consistency, and blood biochemical parameters. A total of 64 pigs (8 pigs × 4 pens × 2 groups) at 7 weeks of age were assigned to control (without EE) and treatment (with EE) groups for four weeks. Pens were the experimental unit for growth, fecal scores, lesion scoring, and behavioral outcomes. Growth and fecal consistency were measured weekly, while ear and tail lesions were scored at the end. Agonistic behavior was quantified using overhead RGB cameras and a YOLOv8-based AI system with high accuracy, mAP50 = 0.953, validated against manual observations, with behavioral outputs aggregated at the pen level from a single representative pen per group. Combined EE reduced lesion severity, lowered free fatty acids, improved fecal consistency, and decreased agonistic behavior, without affecting growth. AI-based monitoring offers a promising tool for quantifying social stress, although further studies with greater pen-level replication are warranted.

## Simple Summary

Group-housed pig production systems can induce considerable stress, particularly during the post-weaning period when separation from the sow and subsequent regrouping disrupt established social hierarchies. Such stressors often precipitate the expression of agonistic behavior, such as aggression, ear biting, and tail biting. Although tail docking is commonly practiced as a preventive measure against tail biting, it neither fully eliminates the behavior nor resolves the underlying social stress, and its associated ethical concerns have led several countries to restrict or ban its use. In this pilot study, rubber sticks and Italian ryegrass hay were provided together as a combined environmental enrichment (EE) package to evaluate their combined effects on pigs’ agonistic behavior. An AI-based system was employed to automatically detect and quantify agonistic behavior. The results demonstrated that pigs receiving EE were engaged in less agonistic behavior. These findings suggest that the combined provision of enrichment materials can substantially enhance the welfare status of pigs. AI-driven behavioral detection, validated against manual scoring by trained observers using the same experimental video recordings, offers a powerful tool for continuous monitoring, providing farmers with timely information on social unrest within groups, thereby enabling more effective intervention strategies to minimize welfare issues and production losses.

## 1. Introduction

Intensification and specialization of pig production, although essential for improving efficiency, simultaneously introduce substantial health and welfare challenges that can ultimately compromise food safety [1]. Among the factors contributing to these challenges, post-weaning periods represent one of the most critical and stressful periods in pigs’ lives, as the abrupt separation from the sow, the sudden transition from milk to solid feed, and the regrouping with unfamiliar pen mates collectively generate physiological and psychological stress that predisposes piglets to a range of detrimental outcomes [2]. Although piglets are commonly weaned at 3–4 weeks of age in commercial production systems, the effects of weaning stress persist into the subsequent nursery phase (5–10 weeks of age), during which piglets continue to undergo physiological and behavioral adaptations while integrating into groups of unfamiliar conspecifics [3,4]. Early weaning at 3 weeks of age has been associated with reduced feeding behavior and lower growth performance during the nursery phase compared with piglets weaned at 4 weeks of age [5]. Moreover, stress-induced elevations in cortisol levels and alterations in immune function not only occur immediately after weaning but also extend beyond the weaning period, thereby adversely affecting production performance and behavioral outcomes during the nursery phase [6,7]. Weaning-related stress further increases aggression and disrupts social interactions, effects that can persist into the nursery phase and ultimately compromise both productivity and animal welfare [3]. This combination of frustration and the unmet motivation for milk-sucking frequently manifests as agonistic behavior [8], which refers to both social aggression (fighting, chasing, and head knocking) and damaging behaviors such as ear and tail biting, with the latter often driven by distinct motivational factors, including frustration and unmet exploratory needs. This behavior not only causes painful injuries [9] but also impair growth and overall productivity [10]. Furthermore, the digestive challenges associated with the abrupt dietary shift often lead to diarrhea, reduced nutrient absorption, and consequently slower growth rates [2].

Tail biting, a prevalent welfare issue in group-housed pigs, is particularly concerning because the resulting open wounds substantially increase the risk of secondary bacterial infections; once toxins produced by these bacteria enter the bloodstream, systemic infection may develop, leading to elevated morbidity and mortality [11,12,13]. Such lesions also contribute to carcass condemnation during preslaughter inspection, thereby increasing production costs [14], while the associated pain, reduced growth, and heightened veterinary and labor demands further burden pig farms [13]. Although tail docking is frequently employed to mitigate tail biting [15], welfare concerns have led to partial or full bans in countries, such as Finland and Sweden [16], and even when implemented, tail docking does not eliminate the behavior entirely [17,18].

Given these limitations, environmental enrichment (EE), such as the provision of straw or manipulable objects, has become increasingly recognized as a practical and welfare-friendly approach to enhancing the physical and behavioral environment of pigs, enabling them to express species-specific exploratory and rooting behavior that may reduce the incidence of agonistic behavior [19,20,21]. Nevertheless, the effectiveness of enrichment strategies must be verified through continuous behavioral monitoring, yet conventional observation methods requiring human presence are laborious, time-consuming, and prone to observer bias while simultaneously altering the pigs’ natural behavior due to the stress caused by human proximity [22]. Although continuous video recording offers an alternative, the manual identification of behavior from recorded footage remains exceedingly time-consuming and inconsistent [23].

The rapid advancement of artificial intelligence (AI), particularly computer vision–based precision livestock farming technologies, presents an opportunity to overcome the above-mentioned challenges; several behaviors, such as feeding, drinking, posture changes, and resting, are now automatically monitored using camera systems and deep learning algorithms [1,24,25,26,27]. You Only Look Once version 8 (YOLOv8), an advanced object-detection model, holds particular promise for automatically identifying agonistic behavior, thereby supporting farmers in improving welfare and production outcomes. Recent YOLOv8-based livestock computer-vision work has demonstrated validated behavior-related outputs in real barn environments [28]. Recent studies have begun exploring the use of accelerometers [29] and AI-enabled camera systems [30,31,32,33,34,35] to detect agonistic behavior in barren environments; however, detection remains challenging due to the brief, complex, and variable nature of such behaviors, which may include fighting, pushing, jumping, and head biting, all lacking a single consistent visual signature [22,36]. Despite substantial progress in precision monitoring technologies [22,34,35,37], further refinement is needed to achieve reliable, real-time detection across diverse housing conditions. Previous studies largely evaluated single EE material; thus, the novelty of the present study lies in testing a combined enrichment strategy (rubber sticks + hay) with multiple welfare outcomes. Therefore, this study aims to evaluate the effects of combined EE on pig production, agonistic behavior, and the automatic detection of agonistic behavior using AI-based computer vision tools.

## 2. Materials and Methods

### 2.1. Test Animals and Experimental Design

**Figure 1:**
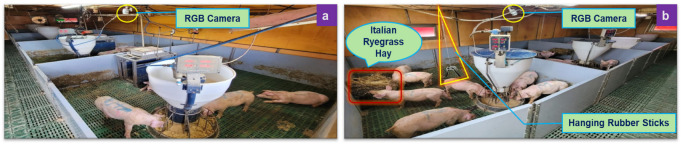
Experimental setup for (**a**) control group (without environmental enrichment) and (**b**) treatment group (with environmental enrichment by rubber sticks and Italian rye grass hay).

A total of 64 weaned pigs (32 gilts and 32 barrows) of (Large White × Landrace) × Duroc cross, 7 weeks of age (average body weight 16.70 kg), were randomly allocated to control (without EE) and treatment groups (with EE) at the Sunchon National University experimental pig farm during the summer months (July–August 2024) in Republic of Korea, with each group comprising 4 pens containing 8 pigs per pen (4 gilts and 4 barrows). Each pen measured 2.35 m × 2.9 m (6.815 m^2^), resulting in an initial stocking density of approximately 2.45 kg/m^2^, and was equipped with an automatic wet–dry feeding system (LFS-120, IONTECH Co., Ltd., Seoul, Republic of Korea) that dispensed feed and water whenever the trough was empty and activated when pigs touching a sensor, providing a feeder space of 0.045 m^2^ per pig and 8 drinker nozzles, thereby ensuring sufficient access for all pigs. A commercially formulated weaner diet was provided to the pigs and was specifically designed to support optimal growth during the nursery phase from 7 to 11 weeks of age. The diet supplied approximately 3350 kcal/kg of digestible energy and 0.90% digestible lysine, thereby meeting established nutritional requirements for nursery pigs. The feed formulation comprised a variety of raw materials, including grains, vegetable peelings, perilla bark, animal fat, molasses, feed salt, limestone, calcium phosphate, magnesium oxide, and essential amino acids (methionine, lysine, tryptophan, and threonine), as well as choline chloride, vitamin–mineral premix, phytase, glucans, and beneficial bacteria. This commercial weaner feed provided adequate energy and essential amino acids without the inclusion of behavioral modifiers. Feed was offered ad libitum to both control and treatment groups to eliminate potential dietary influences on the expression of agonistic behavior. Environmental temperature was maintained at 24 ± 1 °C, with ventilation settings adjusted to provide consistent air exchange across pens, as continuously monitored by a Farm Note system (NareTrends Inc., Bucheon, Republic of Korea), thus supporting both welfare and experimental uniformity throughout the study period. Pigs in the treatment group were provided with rubber sticks (three per pen) and Italian ryegrass hay (1 kg per pen per week). Rubber sticks (approximately 0.30 m in length and 0.009 m in diameter) suspended from chains at back height to allow for chewing and manipulation (Figure 1b); the sticks were inspected daily and replaced if damaged, but were neither cleaned nor rotated. Italian ryegrass hay was supplied in a metal wire basket (0.262 m × 0.18 m × 0.182 m) fixed at back height (Figure 1b), and was partially depleted before weekly replenishment. Throughout the 4-week study, no monopolization or competitive interactions over the enrichment materials were observed, as pigs consistently engaged in exploratory and oral-manipulative behaviors with both rubber sticks and hay, ensuring equitable access and continuous enrichment engagement.

### 2.2. Growth Performance

The body weight of each pig was recorded weekly, and feed supplied was measured at the pen level, with refusals or spillage collected and accounted for, thereby enabling precise calculation of weekly growth performance parameters. Body weight gain, average daily gain, total feed intake, and feed conversion ratio were computed at the end of each week on an as-fed basis. Italian ryegrass hay was provided at 1 kg per treatment pen per week and placed in a designated basket within the pens. Weekly hay intake was determined by weighing the remaining hay at the end of each week, and the consumed amount was included in the total feed intake of the treatment group pigs; however, no correction for differences in nutrient density or dry matter content relative to the basal diet was applied.

### 2.3. Overview of the Computer Vision Model

Images were extracted from video recordings of pigs from separate batches housed in the same experimental facilities described in Section 2.1. A total of 2345 unique images were obtained (Table 1). The image sets were uploaded and annotated in a single Roboflow project, categorizing pigs into three classes of agonistic behavior: aggressive, ear biting, and tail biting, using color-coded bounding boxes to distinguish among behavior classes with the corresponding ethogram presented in Table 2. Each behavior was labeled at the frame level, and consecutive frames were aggregated into behavioral bouts (Table 2).

**Figure 2:**
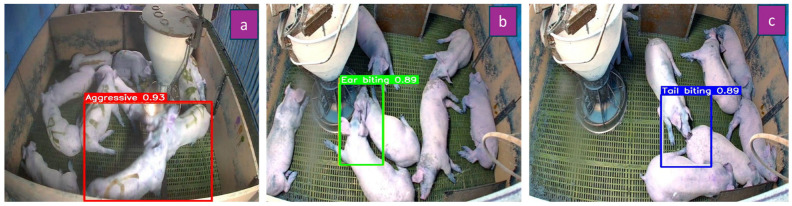
Sample predictions (**a**) aggressive, (**b**) ear biting, and (**c**) tail biting behavior using the agonistic behavior model.

For aggressive interactions, the full body of the physically interacting pigs was annotated, whereas for ear biting and tail biting behaviors, approximately 40% of the body region encompassing the interaction site was considered (Figure 2). These annotations captured critical spatial features, including body position, head contact, and inter-individual distance during interactions. In cases involving multiple pigs, annotation focused on the primary interacting individuals to ensure accurate representation of the agonistic event. The duration thresholds used to define behavioral bouts (≥5 s for aggressive behavior and ≥3 s for ear biting and tail biting) were determined empirically during a pilot observation phase conducted prior to formal data collection. Preliminary inspection of continuous video recordings indicated that very brief contacts (1–2 s) occurred frequently and often reflected incidental contact or exploratory nosing rather than biologically meaningful agonistic interactions. Sustained aggressive encounters typically involved sequences of behaviors such as head knocking, pushing, chasing, or biting that persisted for several consecutive seconds, supporting the use of a longer minimum duration threshold (≥5 s) to reliably capture true aggressive events. In contrast, ear biting and tail biting episodes were generally shorter in duration but repetitive and directed, and a lower threshold (≥3 s) was sufficient to distinguish these behaviors from momentary investigatory contact. These thresholds were finalized through consensus among trained observers during the annotation training phase to ensure consistent and reliable behavioral classification. Subsequent to the annotation process, the datasets were stratified into training, validation, and test subsets, nominally comprising 70%, 20%, and 10% of the total data, respectively; however, the actual partitioning resulted in proportions of 71%, 19%, and 10% for the training, validation, and test datasets, respectively (Table 1). Pre-processing and augmentation were applied prior to dataset download. Prior to model training, all images underwent a standardized pre-processing pipeline designed to minimize variability arising from lighting conditions, camera angle, and pen background structures. Specifically, images were automatically oriented to correct camera rotation, irrelevant background regions were removed through cropping (0–100% horizontal, 0–97% vertical), and all images were resized to 640 × 640 pixels to meet the input requirements of the YOLOv8 framework. Data augmentation was applied exclusively to the training dataset to enhance model robustness under diverse environmental conditions; augmentation strategies included horizontal flipping to enable recognition of behaviors occurring from either side of the pen, brightness adjustments ranging from −15% to +15% to account for lighting fluctuations, Gaussian blurring up to 1.5 px to accommodate motion-induced image blur, and the addition of noise affecting up to 1.76% of pixels for improving tolerance to minor visual disturbances. Augmented images were generated (3336 images), which increased the total training dataset to 5681 images (Table 1). Model training was performed using a pretrained YOLOv8s architecture (yolov8s.yaml) implemented in the Ultralytics YOLOv8 framework (Ultralytics version 8.1.x) on Google Colaboratory, equipped with a Tesla T4 GPU (15 GB VRAM), running Python 3.10.12, and PyTorch 2.2.1. The model was initialized with COCO (Common Objects in Context) pretrained weights and trained for 100 epochs with a batch size of 16, and input images resized to 640 × 640 pixels. Optimization was performed using the AdamW optimizer, automatically selected by YOLOv8’s default configuration, with an initial learning rate of 0.00125 and momentum of 0.9. Default YOLOv8 data augmentation strategies were applied during training, including mosaic augmentation, random horizontal flipping, scaling, translation, and color space augmentations (HSV). A fixed random seed (seed = 42) was used to ensure reproducibility. All remaining hyperparameters followed the Ultralytics YOLOv8 default settings for the specified version. To facilitate efficient learning, transfer learning was applied prior to task-specific training, allowing the model to leverage pre-trained representations of shapes and object boundaries.

**Figure 3:**
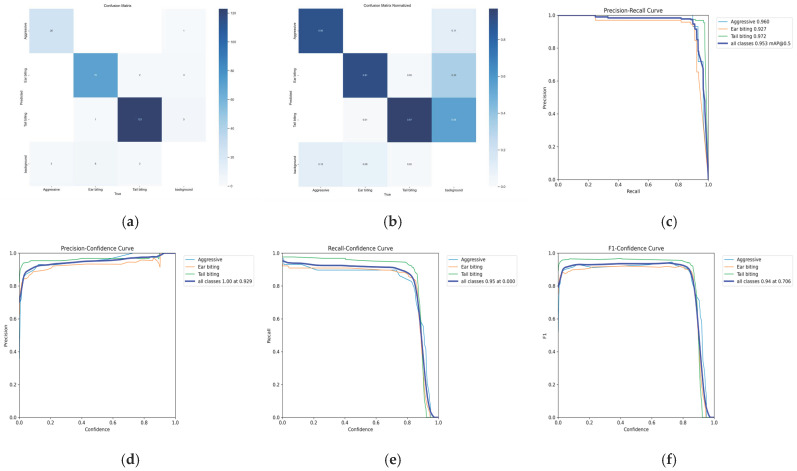
Performance stability of agonistic behavior model, shown by (**a**) confusion matrix, (**b**) normalized confusion matrix, (**c**) per-class precision–recall (AP) curves, and (**d**–**f**) precision, recall, and F1 confidence curves.

There were several advantages to selecting the YOLOv8 model for the automatic detection of agonistic behaviors in pigs. Compared with earlier YOLO versions and conventional computer vision approaches, YOLOv8 offers improved detection accuracy and faster inference speed due to its architectural and functional enhancements [38]. Previous studies have demonstrated the superior performance of YOLOv8, reporting higher precision (87%) compared with YOLOv7 (85.9%) for detecting both common (walking, lying, sniffing, and kneeling) and abnormal (fighting, mounting, and fence climbing) pig behaviors [39]. In particular, the YOLOv8s model employed a CSPDarknet-based backbone [40] for hierarchical feature extraction, a Path Aggregation Network combined with Feature Pyramid Network (PAN-FPN) [41] to integrate multi-scale visual information from both small and large objects, and an anchor-free detection head [40] capable of simultaneously predicting the class and spatial location of agonistic behaviors. The incorporation of a lightweight Cross-Stage Partial Fusion (C2f) feature extraction module [40], along with newly designed loss functions and advanced data augmentation strategies, further improved feature representation, localization precision, and robustness when detecting small objects and complex behavioral scenes [38,42] characterized by frequent occlusion and close animal interactions. These architectural components enabled effective detection of agonistic behavior that varied in size, posture, and interaction dynamics, including scenarios in which pigs were partially occluded by pen equipment or conspecifics. During training, the model learned to localize relevant interaction regions within images and to accurately classify the corresponding behavior patterns, which further enhanced the model’s ability to differentiate among visually similar agonistic behavior even during overlapping or complex movement patterns. During inference, low-confidence detections and overlapping bounding boxes were filtered out to reduce false-positive predictions, such as instances where pigs were in close proximity without exhibiting true agonistic behavior. Importantly, expert observers were not involved in visually selecting, scoring, or subjectively ranking the trained models. Their role was restricted to generating high-quality ground-truth annotations used for supervised training and to independently validating AI-derived outputs, ensuring that model selection remained fully reproducible and free from subjective bias. Model selection was conducted in a fully objective, metric-driven manner based exclusively on quantitative performance on the independent validation dataset. Specifically, the best-performing model was selected based on precision, recall, F1 score, and mean average precision at an intersection-over-union threshold of 0.5 (mAP50). The final model corresponded to the checkpoint achieving the highest overall mAP50 while maintaining balanced precision and recall across all three agonistic behavior classes (aggressive behavior, ear biting, and tail biting), as summarized in Table 3. Figure 2 and Supplementary Videos S1 and S2 demonstrate the predictive capability of the agonistic behavior model in identifying aggressive behavior, ear biting, and tail biting. To further contextualize model robustness given the limited number of test images per class, performance stability of the YOLOv8-based agonistic behavior model was illustrated by the confusion matrix, the normalized confusion matrix, per-class precision–recall (average precision) curves, and precision, recall, and F1 confidence curves (Figure 3). The equations for precision, recall, and F1 score are as follows:

$$Precision=\frac{True\;Positive}{\left(True\;Positive+False\;Positive\right)}$$

$$Recall=\frac{True\;Positive}{\left(True\;Positive+False\;Negative\right)}$$

$$F1\;Score=2\times \frac{\left(Precision\times Recall\right)}{\left(Precision+Recall\right)}$$

### 2.4. Validation of Agonistic Behavior Model

Using a supervised learning framework grounded in expert-annotated image data, the model was systematically trained. All ground-truth annotations were generated by experienced human observers and served as the reference standard for both model training and validation. To ensure the generation of accurate and robust ground-truth annotations, experienced observers meticulously identified and labeled agonistic behavior, including aggressive behavior, ear biting, and tail biting, within extracted video frames. Observers explicitly determined whether each detected behavior represented a correct or incorrect instance of agonistic interaction according to predefined ethograms. Annotations were performed at the frame level (1 frame per second), with each frame labeled independently according to the ethogram and temporal criteria described in Table 2. The model was initialized with pretrained weights derived from the COCO dataset and was subsequently fine-tuned on pig-specific behavioral imagery, with the COCO weights used solely for general visual feature extraction rather than for any pretraining on pig behavior or agonistic interactions, a strategy that enabled the model to learn not only general visual features but also behavior-specific characteristics unique to pigs. Following the model training, the performance of the AI model was systematically compared with manual behavioral observations, with expert human annotations treated as the ground truth against which AI detections were evaluated. Agreement analyses were based on a continuous 24 h video recording; however, the video itself was not treated as a single statistical unit. Instead, it was segmented into multiple independent events and bout-level observational units derived from both manual and AI-based analyses. Trained observers independently analyzed the same 24 h video recordings processed by the AI system and manually quantified agonistic behavior (aggressive, ear biting, and tail biting) in terms of event count, total duration, and duration per bout. Prior to formal data collection, observers participated in a training session for manual behavioral assessment using recorded videos, and inter-observer reliability exceeded 0.8 for agonistic behavior count, total duration, and duration per bout. Frame-level detections generated by the AI model were subsequently converted into biologically meaningful behavioral bouts using a rule-based, threshold-driven algorithm. Bout identification parameters for each behavior class, including minimum bout onset duration and bout termination criteria, are explicitly defined in Table 2. Brief, isolated detections not meeting the minimum temporal thresholds were excluded to reduce noise and false-positive events. Each behavior class was processed independently, and overlapping bouts across different agonistic behaviors (e.g., aggression occurring concurrently with ear or tail biting) were permitted, reflecting the biological reality that multiple agonistic behaviors may co-occur within the same interaction episode. No mutual exclusivity constraints were imposed between behavior classes. Agreement between manual observations and AI-based detection was therefore evaluated across numerous paired behavioral measurements rather than at the video level, using Pearson’s correlation coefficient (r), the intraclass correlation coefficient (ICC; two-way random-effects model with absolute agreement), and Cohen’s kappa (κ) were calculated. For aggressive behavior, event counts demonstrated strong agreement between methods (r = 0.88, ICC = 0.86, κ = 0.80), while total duration showed high consistency (r = 0.85, ICC = 0.83, κ = 0.78), and duration per bout also yielded good agreement (r = 0.82, ICC = 0.80, κ = 0.75). In the case of ear biting behavior, event counts exhibited good agreement (r = 0.84, ICC = 0.82, κ = 0.76), and total duration similarly showed good consistency (r = 0.81, ICC = 0.80, κ = 0.74); however, the agreement for duration per bout was slightly lower, though it remained within an acceptable range (r = 0.79, ICC = 0.78, κ = 0.72). For tail biting behavior, event counts demonstrated good agreement (r = 0.87, ICC = 0.85, κ = 0.79), total duration also showed good agreement (r = 0.83, ICC = 0.81, κ = 0.76), and duration per bout likewise exhibited good consistency (r = 0.80, ICC = 0.79, κ = 0.73). Overall, the strong agreement between AI-derived and human-coded bout metrics confirms that the threshold-based bout-identification algorithm reliably translates frame-level detections into biologically interpretable behavioral outcomes. This expert-driven annotation and validation framework ensures that AI performance was evaluated against biologically meaningful and behaviorally accurate reference data. A schematic description of the bout-identification logic is provided in Supplementary Algorithm S1 to ensure full reproducibility.

### 2.5. Agonistic Behavior Analysis

An RGB camera (PNO-A6081R, Hanwha Vision Co., Ltd., Seongnam, Republic of Korea) with a resolution of 2560 × 1920 was installed on the ceiling of both control (without EE) and treatment (with EE) houses, 2 m above the floor between pens 1 and 2, angled to capture pig activity in pen 2 (Figure 1), with video data stored in AVI format and accessed via cloud storage. Agonistic behavior, which included aggressive, ear biting, and tail biting (Table 2), was analyzed for pen number 2 in both groups, and behavioral outputs were aggregated at the pen level; therefore, behavioral results were interpreted as pilot-scale observations rather than fully replicated treatment effects. Due to access restrictions on the live CCTV feed managed by a third-party provider, all analyses were performed offline. Data extraction and processing were conducted on a desktop computer (Windows 10 (Microsoft, Redmond, CA, USA), Intel^®^ Core™ i5-14400 CPU 2.50 GHz (Intel, Santa Clara, CA, USA), 32 GB RAM, Intel^®^ UHD Graphics 730 GPU (Intel, Santa Clara, CA, USA)) using Python 3.11.6 in Visual Studio Code 1.100.2, with the Ultralytics YOLOv8 framework, PyTorch 2.1.0, and OpenCV 4.8.0. To optimize inference efficiency while preserving sufficient spatial and temporal resolution for behavior detection, video frames were resized from 2560 × 1920 to 800 × 800 pixels and sampled at 1 frame per second (fps). The spatial downscaling was selected to reduce computational load and inference time while retaining adequate visual detail for identifying close-contact agonistic behavior, which involves direct physical interactions between pigs. Temporal sampling at 1 fps was chosen based on preliminary inspections indicating that agonistic behavior typically persists for several consecutive seconds rather than occurring as single-frame events. Object detection was performed using a confidence threshold of 0.45, and outputs were automatically saved in CSV format, including house number (channel 10 = treatment; channel 11 = control), date, timestamp, and per-class detection counts. Another custom Python script implementing a threshold-based algorithm was developed to identify agonistic behavior bouts from processed 1s-resolution datasets. Agonistic behavior bouts were defined in such a way that they started when specific agonistic behavior was observed for a minimum number of consecutive seconds (≥5 s for aggressive, ≥3 s for ear biting and tail biting) and ended when the specific agonistic behavior was absent for a specified duration (≥5 s) (Table 2). Importantly, the same duration criteria were applied consistently to both manual observations and AI-based detections, minimizing methodological bias and ensuring direct comparability between human-coded and automated behavioral outputs. For each bout, starting and end timestamps were recorded, and the difference between these timestamps was the bout duration. Bout frequency was determined by counting the number of events meeting the predefined temporal criteria. Outputs included total bout frequency per day, bout duration (seconds) per day, and duration of each bout (seconds). The use of temporal aggregation criteria reduced the likelihood of systematic undercounting of brief or intermittent detections at 1 fps and ensured consistency between AI-derived outputs and manually annotated behavioral bouts used for validation.

### 2.6. Ear Biting and Tail Biting Lesion Scoring

All pigs were tail-docked prior to the study in accordance with standard commercial management practices at the experimental facility, with approximately two-thirds of the tail removed during the neonatal period, and males were castrated before the start of the experiment. This procedure was applied uniformly across all groups. On the final day, two trained observers evaluated ear and tail biting lesions, following a pre-study training session that achieved interobserver reliability greater than 0.7. Ear lesions were scored according to the system of Diana et al. [43] on a scale of 0–3, where 0 = no lesion; 1 = mild lesions with superficial bites but no blood; 2 = moderate lesions with visible bites, teeth marks, fresh blood, or infection; and 3 = partial or total loss of the ear. Scores for both ears of each pig were averaged. Tail lesions were assessed using the scoring system of Kritas and Morrison [44] on a scale of 0–4, where 0 = no evidence of tail biting; 1 = healed or mild lesions; 2 = chewing or puncture wounds without swelling; 3 = chewing or puncture wounds with swelling and signs of potential infection; and 4 = partial or total loss of the tail. For statistical analysis, lesion scores were first calculated at the individual pig level and subsequently averaged within each pen to obtain pen-level mean lesion scores, which were used as the experimental unit to account for clustering of pigs within pens.

### 2.7. Blood Biochemical Parameters

A total of 24 blood samples were collected on the final day of the study from 24 pigs (12 pigs per group), corresponding to 3 pigs randomly selected from each pen (3 pigs × 4 pens × 2 groups). Blood samples were obtained via jugular venipuncture, with approximately 5 mL collected from each pig into heparinized vacuum tubes and immediately stored in an icebox. Sampling was conducted in the morning hours (10:00–11:00) to minimize diurnal variation, and pigs were not fasted prior to sampling, reflecting standard commercial management conditions. Plasma was separated by centrifugation and stored at −20 °C until biochemical analysis. Blood biochemical parameters, including glucose, creatine kinase, lactate dehydrogenase, free fatty acids, and cortisol, were analyzed by Global Clinical Central Lab, Yongin, Republic of Korea.

### 2.8. Fecal Scores

Initial fecal scores were recorded prior to the start of the study, and fecal scores were subsequently assessed each week to monitor the incidence of diarrhea. Fecal consistency was evaluated by observing feces present in the four corners of each pen. When feces were unevenly distributed or absent in one or more corners, scoring was based on the available fecal material, and if no fresh feces were present in any corner at the time of observation, the score from the nearest subsequent observation period on the same day was used. The average fecal score per pen was then calculated and used for statistical analysis. Prior to fecal scoring, observers participated in a training session to standardize scoring procedures, achieving an interobserver reliability greater than 0.9. Due to the visible presence of EE materials, blinding of observers to treatment allocation was not feasible; however, scoring criteria were strictly predefined and applied consistently across all pens to minimize observer bias. Fecal scores were defined as follows: 0 = normal, dry, and firm feces; 1 = soft and moist feces; 2 = mild diarrhea with soft, unformed feces; and 3 = severe diarrhea with watery feces [45].

### 2.9. Statistical Analysis

IBM SPSS Statistics (version 17.0; IBM Corp., Armonk, NY, USA) was employed for all statistical analyses. To appropriately account for the hierarchical structure of the data and the presence of repeated measurements over time, linear mixed-effects models were applied for outcomes measured longitudinally for growth performance parameters, agonistic behaviors, and fecal score of pigs. Ear biting and tail biting lesion scores and blood biochemical parameters were measured using independent samples *t*-tests.

## 3. Results

### 3.1. Growth Performance

Body weight increased progressively over the 4-week study period in both control (without EE) and treatment (with EE) groups (Table 4), with no significant main effect of group, indicating that mean body weight did not differ between treatments when averaged across weeks, whereas a strong effect of week (*p* < 0.001) reflected normal growth over time. However, the presence of a significant group × week interaction (*p* = 0.036) indicates that temporal patterns of body weight gain differed between groups. Consistent with this finding, average daily body weight gain (ADG) varied significantly across weeks (*p* < 0.001) in the absence of a main group effect, while a significant group × week interaction (*p* = 0.021) revealed week-dependent differences in growth rate, with enriched pigs exhibiting higher ADG in weeks 2 and 4 but lower ADG in week 3, suggesting a transient modulation of growth dynamics associated with EE. Average daily feed intake increased gradually over time in both groups, as indicated by a significant effect of week (*p* = 0.015), whereas neither the group effect nor the interaction term reached significance, demonstrating that EE did not substantially alter overall feed intake patterns. Feed conversion ratio (FCR) was significantly influenced by week (*p* < 0.001), reflecting age-related changes in feed efficiency, and although no main group effect was detected, a significant group × week interaction (*p* = 0.009) was observed, driven primarily by a lower FCR in the EE group during week 4, indicating improved feed efficiency at the later stage of the experimental period under enriched conditions. These results demonstrate that while EE did not affect cumulative growth performance, it modulated week-specific growth efficiency, as evidenced by consistent group × week interactions for body weight, ADG, and FCR.

### 3.2. Agonistic Behavior of Pig

EE was associated with a marked reduction in agonistic behavior relative to the control condition (without EE) (Table 5), as evidenced by consistently lower daily aggressive counts across all study weeks (group effect: *p* = 0.007), alongside a general decline over time in both groups (week effect: *p* = 0.005), in the absence of a significant group × week interaction, indicating a stable treatment effect throughout the study period. Similarly, the total duration of aggressive behavior per day was substantially reduced in enriched pens (*p* < 0.001) and decreased progressively across weeks (*p* < 0.001), while aggressive duration per bout was also shorter in the EE group (*p* = 0.001) and varied temporally (*p* < 0.001), with a near-significant interaction (*p* = 0.054) suggesting an increasing magnitude of the enrichment effect at later stages. Although ear biting counts per day were numerically lower in enriched pens, the lack of a significant group effect indicates that enrichment did not consistently reduce event frequency; however, both ear biting duration per day (*p* < 0.001) and duration per bout (*p* = 0.003) were significantly reduced, demonstrating a decrease in cumulative engagement and bout persistence. Tail biting behavior was likewise attenuated by enrichment, with significant reductions in daily event counts (*p* < 0.001), total duration per day (*p* < 0.001), and duration per bout (*p* = 0.014), accompanied by a modest temporal effect on event frequency (*p* = 0.033) and a marginal group × week interaction (*p* = 0.056), collectively indicating that the combined provision of rubber sticks and Italian ryegrass hay consistently reduced the expression and severity of agonistic interactions across the study period.

**Figure 4:**
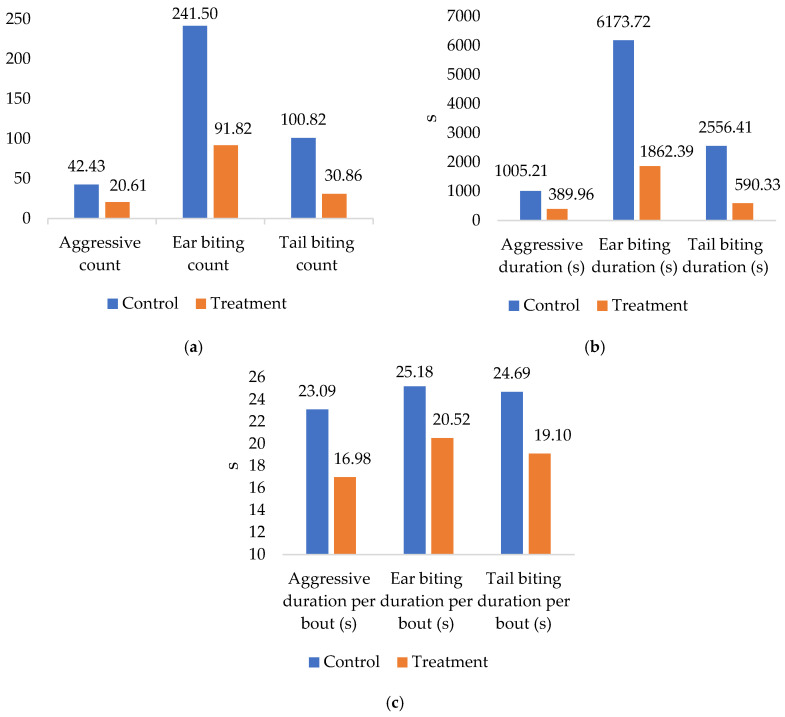
Overall agonistic behavior in control (without environmental enrichment) and treatment groups of pigs (with environmental enrichment by rubber sticks and Italian ryegrass hay). (**a**) Daily counts of aggressive, ear biting, and tail biting behavior; (**b**) total daily duration (s) of aggressive, ear biting, and tail biting behavior; and (**c**) mean duration per bout (s) of aggressive, ear biting, and tail biting behavior.

To more robustly evaluate temporal trends in agonistic behavior, a linear mixed model was applied using daily observations across the 28-day period (Table 6). This analysis revealed a strong main effect of treatment, with enriched pigs exhibiting significantly lower overall aggressive counts, total aggressive duration per day, and aggressive duration per bout compared with control pigs (*p* < 0.001 for all parameters). A significant main effect of day was also observed for all aggressive and tail biting measures (*p* < 0.001), indicating a progressive decline in agonistic behavior over time across both groups. Importantly, significant treatment × day interactions were detected for aggressive counts, aggressive duration, and tail biting measures (*p* < 0.001), demonstrating that the magnitude of behavioral reduction over time was greater in enriched pigs than in controls. For ear biting behavior, although the overall frequency did not differ significantly between groups, significant effects of day and treatment × day interaction were observed for ear biting duration and duration per bout (*p* ≤ 0.008), indicating that enrichment reduced the persistence and intensity of ear biting episodes over time. These findings confirm that EE not only reduced the overall level of agonistic behavior but also accelerated its decline across the study period. Overall agonistic behavior in the control and treatment groups of pigs is shown in Figure 4.

**Figure 5:**
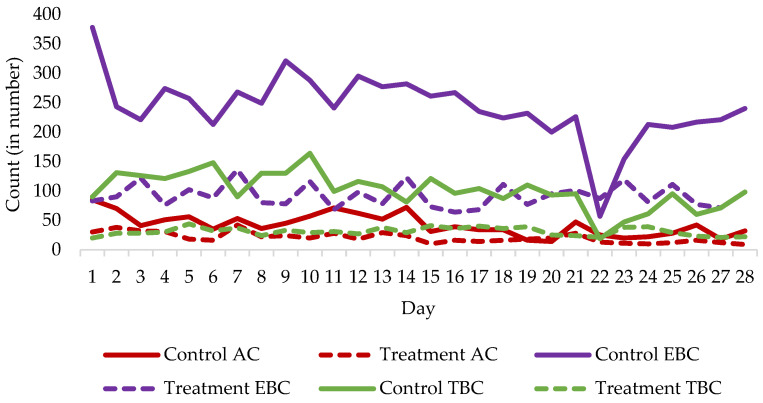
Aggressive count (AC), ear biting count (EBC), and tail biting count (TBC) in control (without environmental enrichment) and treatment groups of pigs (with environmental enrichment by rubber sticks and Italian ryegrass hay) from 1st to 28th days of study.

Daily patterns of agonistic behavior differed markedly between control (without EE) and treatment pigs (with EE) over the 28-day study period (Figure 5). Treatment pigs consistently exhibited lower counts of all agonistic behavior, with reduced fluctuations compared to control pigs, which showed higher and more variable daily values. Aggressive behavior in control pigs remained elevated on most days, whereas treatment pigs maintained consistently lower and more stable counts. A similar trend was observed for ear biting, with control pigs exhibiting pronounced peaks, particularly during the early and mid-study period, while treatment pigs displayed consistently lower counts. Tail biting counts also differed between groups, with treatment pigs showing lower and stable daily values, in contrast to higher and more variable counts in controls. Overall, the provision of EE effectively reduced daily agonistic behavior counts, and a general decreasing trend in all behavior was observed over the course of the study in both groups.

### 3.3. Ear Biting and Tail Biting Score

Table 7 indicates that pigs in the treatment group (with EE) exhibited significantly lower ear biting scores compared to control pigs (without EE). A similar pattern was observed for tail biting scores, with treatment pigs showing significantly lower values than control pigs.

### 3.4. Blood Biochemical Parameters

Analysis of blood biochemical parameters of pigs associated with stress showed that free fatty acid levels were significantly lower in the treatment group (with EE) than in the control group (without EE) (Table 8). Lactate dehydrogenase concentrations were also lower in treatment group pigs compared with controls, although this difference did not reach statistical significance (*p* = 0.096), indicating a tendency toward reduced levels in the EE group.

### 3.5. Fecal Score of Pig

Fecal consistency differed significantly between the control group (without EE) and the treatment group (with EE) across the study period (Table 9), as evidenced by a significant main effect of group (*p* = 0.004), indicating an overall influence of EE on fecal score. In addition, a significant effect of week was detected (*p* < 0.001), demonstrating pronounced temporal changes in fecal consistency as pigs progressed through the nursery phase. Moreover, a significant group × week interaction was observed (*p* = 0.006), indicating that the trajectory of fecal score over time differed between the two groups. From week 1 onward, fecal scores declined substantially in both groups, with the treatment group exhibiting consistently lower fecal scores during weeks 2 to 4. This pattern suggests that pigs housed in enriched pens experienced a more rapid stabilization of fecal consistency over time compared with pigs in non-enriched pens. Collectively, these results indicate that the provision of EE with rubber sticks and Italian ryegrass hay was associated with improved fecal consistency during the later stages of the study period, reflecting a potential beneficial effect of enrichment on gastrointestinal function as pigs adapted to post-weaning conditions.

## 4. Discussion

This pilot study evaluated the effects of EE using rubber sticks and hay on the growth performance of pigs, and overall, EE had no effect on growth performance. It may reflect the time required for pigs to adapt in a new environment enriched with novel materials, particularly as weaning involves simultaneous stressors, including separation from the sow, abrupt dietary changes, and regrouping with unfamiliar penmates. The previous literature presents inconsistent findings regarding the impact of EE on growth performance. Positive effects have been reported, such as increased average daily gain with play objects [46] and higher growth rates with longer straw-based enrichment periods [47]. Beattie et al. [48] similarly observed improved performance, but only during the finishing phase. Oostindjer et al. [49] further demonstrated that relocating piglets from barren pre-weaning conditions to enriched post-weaning environments with straw, wood shavings, peat, and branches enhanced body weight and feed efficiency in the second week after weaning, suggesting that both EE duration and timing relative to pig age are influential. However, several studies, including those by Beattie et al. [50], Oliveira et al. [51], Caldara et al. [52], Fu et al. [53], Nannoni et al. [54], Klont et al. [55], Bulens et al. [56], and Wen et al. [57] report no effect of EE on growth performance. The findings of the present study align with this group of studies, indicating a limited overall impact of EE on growth performance parameters. Rather than merely reiterating previous observations, this study reveals that a combined enrichment approach involving hanging rubber sticks and wall-mounted hay in slatted-floor housing systems preserves production performance and promotes improved behavioral and welfare outcomes.

EE using rubber sticks and hay markedly reduced agonistic behavior count (except ear biting count), both in their duration and duration per bout. Pigs in the EE group spent considerable time interacting with the enrichment materials for sucking, chewing, and playing with the rubber sticks, and chewing and ingesting the hay. Pigs naturally prefer materials that are chewable, deformable, rootable, and destructible [58], and when enrichment fulfills these criteria, they remain engaged for longer periods [59,60]. Rooting and foraging are highly motivated natural behaviors, and pigs want to exhibit this behavior even if it does not give energy or improve body growth [61,62]. Demand functions indicate the extent to which farm animals are willing to work to obtain specific resources, such as feed or opportunities for rooting and foraging [63]. When an animal continues to express a particular behavior despite increasing costs in terms of effort or work, this persistence reflects the high motivational importance of that behavior. For example, pigs are willing to exert greater effort to access feed [64], demonstrating that feed represents an essential resource with a high priority. In the present pilot study, hay was provided in racks, and pigs exhibited foraging behavior; however, the provision of such enrichment is challenging in slatted-floor housing systems, where the use of loose substrates is limited. The combination of hanging rubber sticks and hay in racks increased pigs’ interaction time around the enrichment area, which likely contributed to reducing agonistic behavior. Pigs exhibit strong motivation for exploratory behavior, and when opportunities for such behavior are limited, they are more prone to aggression [65] and, if their behavioral needs remain unmet, lead to ear and tail biting [66]. Frustration arising from insufficient exploratory opportunities may escalate the frequency and duration of agonistic behavior. The pigs’ clear preference for the provided EE materials likely explains the reduced agonistic behavior, except for the ear biting count observed in the EE group. Additionally, hay offers some nutritional benefits that may reduce the risk of gastric ulcers, similar to grass silage [67]. Thus, the combined use of rubber sticks and hay better satisfies pigs’ behavioral needs, contributing to lower levels of agonistic behavior. Several previous studies have explored pigs’ preferential responses to various EE materials and investigated how these preferences modulate the expression of agonistic behavior. Chou et al. [68] reported that pigs preferred rubber toys placed on the floor over fixed wooden sticks. Beattie et al. [50] found that peat and straw in racks reduced aggressive behavior and eliminated tail biting, whereas Beattie et al. [48] reported only reductions in aggressive duration. Schaefer et al. [69] observed fewer aggressive acts when car tires were used as enrichment. Bolhuis et al. [60] and Wen et al. [57] showed that straw or mixed bedding (straw, peat, wood shavings, jute bags, and branches) reduced aggressive behavior. Rosvold et al. [70] reported that while straw and hay as bedding did not reduce aggression, straw reduced both ear and tail biting, and hay reduced tail biting only, and that agonistic behavior decreased with age. The present findings align with Beattie et al. [50], Schaefer et al. [69], Bolhuis et al. [58], and Wen et al. [57] for reductions in aggressive behavior; Beattie et al. [48] for shorter aggressive duration; and Rosvold et al. [70] for reduced tail biting and the decreasing trend of aggressive behavior and tail biting over time. However, the result of this study differs from that of Rosvold et al. [70] regarding aggressive behavior. This discrepancy may be attributable to the fact that, in the present study, hay was offered in a basket rather than as bedding material and was supplied in combination with hanging rubber sticks, thereby creating a distinctly different EE configuration.

EE reduced ear and tail biting scores, indicating lower lesion severity in treated pigs. Despite minimal effects on ear biting frequency, reduced daily duration and bout length suggest that enrichment attenuated the persistence and intensity of biting episodes, which may be more closely linked to tissue damage and thus contribute to the lower ear biting scores observed in enriched pigs. EE materials are typically provided to stimulate exploratory, manipulative, and foraging-related behavior; therefore, pigs are expected to devote substantial time to interacting with such materials rather than agonistic behavior toward conspecifics. Pigs initially demonstrated a high level of curiosity toward both the rubber sticks and hay; however, as the study progressed, they gradually became habituated to these materials, suggesting that enrichment-induced behavioral engagement is influenced by both novelty and duration of exposure. The rubber sticks offered multiple functional properties; they were playable, suckable, chewable, and even destructible, which likely supported species-typical oral manipulation. Pigs may have performed a sequence of manipulative behaviors involving touching, grasping, chewing, and ultimately ingesting the hay. Species-specific enrichment is essential to facilitate expression of positive social behavior, and pigs are known to prefer materials that are chewable, destructible, ingestible, and manipulatable through jaw movement [59,71]. Thus, the combination of rubber sticks and hay likely fulfilled these behavioral motivations, thereby reducing the incidence of agonistic behavior and the resulting ear and tail biting lesions. This behavioral preference is supported by Chou et al. [72], they observed that pigs spent considerably more time interacting with a straw rack, through touching, grasping, and chewing, than with a hanging rubber toy. Additional studies have similarly reported a strong preference for straw, either as bedding or in racks [60,73,74,75]. Several previous studies have demonstrated that species-specific EE can attenuate both ear biting and tail biting lesions. Zonderland et al. [76] found that providing straw on the floor substantially reduced the severity of tail wounds compared with metal chains or rubber hoses; moreover, supplying straw twice daily minimized moderate tail lesions more effectively than straw racks or other enrichment devices. Rosvold et al. [70] further observed that straw and hay used as bedding material decreased the proportion of pigs with tail biting marks. Nannoni et al. [54] reported that hanging metal chains reduced tail biting lesions relative to wood logs, presumably because chains allow for more continuous oral manipulation. In contrast, Fu et al. [53] demonstrated that the use of a toy as EE reduced ear lesions but did not significantly reduce tail biting damage. Overall, the findings of this study align with those of Zonderland et al. [76], Rosvold et al. [70], and Nannoni et al. [54] regarding tail biting lesions, and with Fu et al. [53] for ear biting lesions.

EE using rubber sticks and hay resulted in a measurable decrease in circulating free fatty acids and showed a tendency to reduce lactate dehydrogenase concentrations (*p* value = 0.096); however, EE did not exert detectable effects on blood glucose, creatine kinase, and cortisol levels. Although pigs provided with EE exhibited significantly lower circulating free fatty acid concentrations, this metabolic difference was not accompanied by a significant main effect of treatment on average daily body weight gain. This indicates that the observed reduction in free fatty acids likely reflects short-term changes in energy mobilization or physiological stress rather than enhanced cumulative growth performance. Free fatty acids are sensitive indicators of acute metabolic demand and stress-related lipid mobilization, whereas average daily body weight gain integrates longer-term nutrient utilization and growth. Allowing animals to perform motivated behaviors may alleviate behavioral signs of stress despite minimal variation in physiological stress indicators [77], highlighting the importance of intrinsic behavioral expression in modulating stress independently of endocrine responses. The reduction in agonistic behavior observed in enriched pigs may therefore have lowered the energetic costs associated with social conflict without necessarily translating into detectable differences in overall growth rate. The availability of enrichment materials likely encouraged pigs to allocate more time to play behavior involving the rubber sticks and to manipulative exploration of the hay, thereby reducing the frequency and intensity of energetically demanding agonistic behavior. Consequently, reduced physical conflict may have contributed to the lower levels of free fatty acids observed in enriched pigs, as mobilization of fatty acids typically increases during periods of elevated activity or stress condition [78].

Alterations in circulating metabolites can provide biochemical signals to the central nervous system, thereby influencing behavioral expression [79], which further supports the possibility of behavior–physiology interactions in enriched environments. Several previous studies have demonstrated the influence of EE on the blood biochemical parameters of pigs. Peeters et al. [47] reported that straw bedding as EE had no effect on blood glucose, lactate, fatty acids, and cortisol in pigs. They also observed that blood creatine kinase did not differ between pigs of EE and barren conditions when straw was provided for only four weeks, whereas a reduction in this enzyme was detected when enrichment was continued for six weeks, highlighting the importance of EE duration in eliciting physiological changes. Similarly, Quesnel et al. [80] found no influence of EE using hanging oak pieces and straw pellets on blood glucose, lactate, and fatty acids.

Cortisol, a key biomarker of stress measurable in blood, saliva, urine, feces, and milk [81], generally increases during acute stress [82], whereas hair cortisol is considered a reliable indicator of chronic stress exposure [83]. Nannoni et al. [54] showed that hanging chains, wood logs, and edible blocks as EE had no effect on hair cortisol concentrations. Casal et al. [84] further demonstrated that pigs housed with sawdust, natural hemp rope, and rubber showed reduced hair cortisol compared with pigs kept in barren conditions, but only after eight weeks of enrichment exposure; no effects were observed at four weeks. Taken together, these findings imply that short-term enrichment, such as the four-week period in the present study, may be insufficient to alter blood cortisol levels. Overall, the results of this study correspond with the findings of Peeters et al. [47] and Quesnel et al. [80] for blood glucose and lactate dehydrogenase, and with Peeters et al. [47] for cortisol, but deviate regarding free fatty acid concentrations. Such discrepancies may be attributed to differences in enrichment type, spatial arrangement, combinations of materials, and the age of the pigs, all of which may influence the extent to which EE modulates metabolic parameters.

Post-weaning diarrhea is widely recognized as one of the most prevalent challenges encountered by pig farmers [85], largely because the transition from the pre-weaning to the post-weaning environment exposes piglets to multiple concurrent stressors, including abrupt environmental change, separation from the sow, a dietary shift from easily digestible milk to solid feed, and regrouping with unfamiliar piglets, which collectively reduce feed intake and alter fecal consistency [4]. In the present study, pigs housed in the EE exhibited consistently lower fecal scores compared with pigs maintained in barren conditions, with this pattern becoming increasingly evident during the second, third, and fourth weeks of the study. The provision of rubber sticks and hay may have facilitated pigs’ adaptation to the new environment by encouraging exploratory and manipulative behavior that may contribute to stress mitigation. Furthermore, the weekly provision of fresh hay likely stimulated foraging activity and may have contributed to improved gastrointestinal function, thereby reducing fecal scores in the EE group.

Hay is known to contain substantial amounts of crude fiber [86] and insoluble dietary fiber [87], the latter playing a crucial role in increasing fecal bulk and accelerating gastrointestinal passage rate; these changes reduce the window of opportunity for pathogenic microorganisms to proliferate within the gut [88]. Insoluble fiber also promotes the growth of beneficial bacteria in the large intestine, enhancing short-chain fatty acid production, limiting adhesion and multiplication of enteric pathogens, such as *Escherichia coli*, and ultimately contributing to improved gut health [88,89,90,91]. Thus, the inclusion of hay as part of EE may have improved gastrointestinal stability and contributed to the observed reduction in fecal scores, thereby lowering the risk of post-weaning diarrhea. Additionally, hay contains measurable concentrations of zinc [92], a mineral with well-established antimicrobial and gut health-promoting properties. Zinc can reduce bacterial adhesion to epithelial cells, thereby exerting antibacterial effects [93], and can enhance intestinal morphology by upregulating the expression of tight junction proteins, which reduces intestinal permeability and improves barrier integrity [93,94]. These actions of zinc may further contribute to the observed improvements in gut health, as reflected by the reduced fecal scores in enriched pigs. Several previous studies have also highlighted the positive effects of EE on gut health and diarrhea incidence. Lee et al. [46] reported that providing play objects during the first four weeks post-weaning reduced fecal scores and consequently decreased the incidence of diarrhea. Oostindjer et al. [49] demonstrated that piglets moved from barren pre-weaning conditions to enriched post-weaning environments (containing wood shavings, peat, straw, and branches) exhibited shorter durations of post-weaning diarrhea compared with piglets housed in barren conditions throughout. Pluske et al. [95] found that straw bedding increased microbial diversity in the large intestine of enriched pigs, a factor that may contribute to the reduced prevalence of diarrhea. Wen et al. [57] observed that bedding materials, such as straw, moist peat, wood shavings, jute bags, and broom branches, increased beneficial bacteria and reduced the abundance of the genus Enterococcus in pre-weaning pigs, which may contribute to a lower incidence of diarrhea, although this effect was not sustained post-weaning. The studies by Oostindjer et al. [49], Pluske et al. [95], and Wen et al. [57] converge on the conclusion that EE can attenuate diarrheal incidence, a pattern that necessarily corresponds with lower fecal scores in enriched pigs. Consistent with this broader evidence base, the present study directly corroborates the observations of Lee et al. [46] by demonstrating reduced fecal scores in pigs provided with EE, while also indirectly reflecting the diarrhea-reducing effects described by Oostindjer et al. [49], Pluske et al. [95], and Wen et al. [57], given that diminished diarrhoeal incidence ultimately manifests as lower fecal scores.

This study provides pilot-scale evidence and novel insights demonstrating that a strategically designed combination of EE using rubber sticks and hay, adapted for slatted-floor housing, can influence agonistic behavior, lesion outcomes, and selected physiological indicators in group-housed pigs, thereby improving welfare by reducing agonistic behavior and stress-related outcomes without compromising productivity. However, several limitations warrant consideration. The short study duration restricted the opportunity for pigs to fully adapt to the enriched environment and prevented assessment of age-related changes in agonistic behavior or the development of chronic stress, which would typically be reflected in long-term indicators, such as hair or blood cortisol. The use of a single combination of EE materials makes it impossible to disentangle the specific contribution of rubber sticks versus hay, and potential synergistic or antagonistic interactions among different EE materials remain unexplored. The YOLOv8-based system demonstrated the capability to automatically detect agonistic behaviors in pigs under conditions of partial occlusion caused by pen equipment; however, its performance may be compromised when pigs are severely obscured by such equipment, representing a limitation of the automatic agonistic behavior detection model employed in this pilot study. The relatively small sample size and environmentally controlled housing further limit the external validity of the findings, particularly for commercial systems that vary widely in stocking density and management practices. All pens of the same treatment were housed within the same environmentally controlled house (control pens in one house, treatment pens in another), which confounds treatment with house. Therefore, pens cannot be treated as fully independent replicates across houses, and this limitation may influence the generalizability of the results. The use of a single pen per group for agonistic behavioral observations further limit the generalizability of the findings and may introduce pseudo-replication. Similarly, validation of the AI-based agonistic behavior detection model was conducted using a single continuous 24 h video recording, and although agreement analyses were performed on multiple event and bout-level observations extracted from this recording, the results primarily reflect within-system agreement rather than between-system generalizability. In addition, all pigs were tail-docked, which may have reduced the prevalence and severity of tail lesions; thus, the effects of EE on tail biting should be interpreted within the context of docked pigs. Although blood metabolites and fecal scores were measured, other welfare-relevant parameters, such as immune function, gastrointestinal and fecal microbiota profiles, and gastric lesion assessments were not included. Fresh hay was supplied weekly rather than daily, which may not have fully satisfied pigs’ continuous motivation to perform foraging behavior and may have constrained sustained engagement with the enrichment materials. With respect to the AI-based behavioral monitoring framework, despite the strong agreement between automated detections and manual observations, and the improved precision achieved through an augmented training dataset including aggressive, ear biting, and tail biting classes, this pilot study was limited by the relatively small size of the training dataset.

Given these limitations, this study represents a pilot-scale investigation, and future studies incorporating multiple pens for agonistic behavior observations, multiple independent recording days, and pens for AI model validation, a higher number of training images, and additional houses for each treatment are necessary to confirm and extend these findings while avoiding pseudo-replication. Future research should extend EE studies across the full production cycle, from weaning to slaughter, to elucidate the long-term impacts of enrichment on growth performance, health, and welfare. Systematic evaluation of a broader range of EE materials, both individually and in combination (rubber, plastic, wood, straw, hay, ropes, and edible blocks), and deployed in varied spatial configurations, such as hanging, wall-mounted, or floor-based, would clarify material and position-specific preferences and their capacity to mitigate agonistic behavior. More frequent replenishment of hay could potentially enhance behavioral engagement and welfare outcomes. Integrating additional welfare-related measurements, including gut and fecal microbiota profiling, intestinal morphology, stress-responsive gene expression, and immune biomarkers, would further deepen understanding of the physiological pathways through which EE exerts its effects. With respect to blood biochemical parameters, future studies should incorporate larger and more balanced sampling schemes across pens and housing units to reduce the influence of clustering and improve statistical power, allowing metabolic responses to be interpreted with greater confidence. Conducting trials under commercial farm conditions with diverse stocking densities and flooring systems would strengthen the external validity and practical applicability of the findings. Moreover, reporting effect sizes and confidence intervals alongside *p*-values will be critical for aligning physiological interpretation with the strength and precision of statistical evidence, particularly for metabolic indicators derived from limited sample sizes. Future research is needed to enhance the robustness of agonistic behavior detection under severe occlusion by employing multi-camera setups positioned at different locations within the pen, expanding and diversifying training datasets, and integrating complementary sensing technologies, such as depth cameras, wearable accelerometers, and acoustic sensors. Furthermore, research aimed at identifying optimal renewal intervals, quantities, and spatial placement of EE materials will be essential to ensure sustained engagement and effective reduction in agonistic behavior in group-housed pigs.

## 5. Conclusions

This pilot-scale study demonstrates that EE using hanging rubber sticks and Italian ryegrass hay in a basket can reduce agonistic behavior and associated lesion severity in group-housed pigs under controlled conditions, without adversely affecting overall growth performance. Enriched pigs exhibited consistently lower frequencies and durations of agonistic behavior except ear biting frequency, accompanied by reduced ear and tail lesion scores, indicating improved welfare. Physiological responses provided partial support for reduced stress, as evidenced by lower circulating free fatty acid concentrations, although other blood biochemical indicators and growth metrics were largely unaffected, warranting cautious interpretation. The AI-based computer vision system showed strong agreement with manual observations, supporting its utility for objective quantification of agonistic behavior in experimental settings. The combined application of EE and AI-assisted behavioral monitoring is promising for enhanced welfare assessment in group-housed pigs; however, these findings should be regarded as preliminary and require validation under commercial production conditions using larger sample sizes, multiple pens, and independent housing units. Future research should extend this approach by evaluating multiple enrichment materials across the full production cycle, from weaning to marketing age, while integrating economic analyses.

## Figures and Tables

**Table 1:** Image dataset information.

Dataset	Raw Images	Percentage (%) of Total Raw Data	Augmented Images	Total Images Used	Percentage (%) of Total Images Used
Training	1668	71	3336	5004	88
Validation	446	19	0	446	8
Test	231	10	0	231	4
Total	2345	100	3336	5681	100

**Table 2:** Ethogram of agonistic behavior with temporal bout criteria and labeling scheme.

Behavior	Characteristics	Temporal Criteria (Bouts)	Label Type
Aggressive	Forcefully head-knocking, body-knocking, chasing, and mounting the conspecific	Bout starts when behavior occurs for ≥5 s; ends when absent for ≥5 s	Frame-level, aggregated into bouts
Ear biting	Sucking, biting, and chewing of the ear of a conspecific	Bout starts when behavior occurs for ≥3 s; ends when absent for ≥5 s	Frame-level, aggregated into bouts
Tail biting	Repetitive sucking, biting, and chewing of the tail of conspecifics	Bout starts when behavior occurs for ≥3 s; ends when absent for ≥5 s	Frame-level, aggregated into bouts

**Table 3:** Performance metrics of the agonistic behavior model.

Class	Images	Precision	Recall	F1 Score	mAP50
Aggressive	29	0.990	0.897	0.941	0.960
Ear biting	77	0.943	0.896	0.919	0.927
Tail biting	126	0.963	0.948	0.958	0.972
All	231	0.967	0.914	0.939	0.953

**Table 4:** Growth performance of pigs.

Parameter	Week	Group		*p*-Value		
		Control (Mean)	Treatment (Mean)	Group	Week	Group × Week
Body weight (kg)	0 *	16.70	16.68	0.603	<0.001	0.036
	1	23.47	22.88			
	2	29.69	29.63			
	3	35.35	34.02			
	4	38.49	39.09			
Average daily body weight gain (kg)	1	0.97	0.89	0.605	<0.001	0.021
	2	0.89	0.97			
	3	0.81	0.63			
	4	0.45	0.73			
Average daily feed intake (kg)	1	1.36	1.38	0.145	0.015	0.686
	2	1.39	1.60			
	3	1.48	1.51			
	4	1.63	1.69			
Feed conversion ratio	1	1.42	1.62	0.783	<0.001	0.009
	2	1.58	1.61			
	3	1.83	2.77			
	4	3.71	2.36			

Control—no environmental enrichment, Treatment—environmental enrichment by rubber sticks and Italian ryegrass hay, 0 *—initial day of the study.

**Table 5:** Weekly mean values of agonistic behavior in control (without environmental enrichment) and treatment (with environmental enrichment) pigs.

Parameter	Week	Group		*p*-Value		
		Control (Mean)	Treatment (Mean)	Group	Week	Group × Week
Aggressive count per day	1	59.70	31.03	0.007	0.005	0.260
	2	56.60	23.09			
	3	30.85	17.42			
	4	25.18	10.67			
Aggressive duration (s) per day	1	1551.11	751.09	<0.001	<0.001	0.498
	2	1228.46	492.04			
	3	745.55	200.95			
	4	503.68	110.12			
Aggressive duration per bout (s)	1	27.71	25.13	0.001	<0.001	0.054
	2	22.31	21.59			
	3	23.77	11.19			
	4	18.77	10.08			
Ear biting count per day	1	325.64	137.15	0.563	0.071	0.151
	2	309.94	96.07			
	3	297.88	61.82			
	4	149.29	59.49			
Ear biting duration (s) per day	1	7427.44	1877.23	<0.001	0.255	0.271
	2	6338.16	1866.04			
	3	6088.41	1847.22			
	4	4888.12	1852.09			
Ear biting duration per bout (s)	1	28.38	19.10	0.003	0.696	0.253
	2	22.65	21.11			
	3	25.39	21.78			
	4	24.26	20.15			
Tail biting count per day	1	116.95	31.38	<0.001	0.033	0.056
	2	117.52	29.29			
	3	105.04	34.56			
	4	64.04	27.55			
Tail biting duration (s) per day	1	2967.39	606.58	<0.001	0.173	0.245
	2	3023.09	589.53			
	3	2755.17	632.46			
	4	1466.37	525.93			
Tail biting duration per bout (s)	1	24.54	19.19	0.014	0.956	0.816
	2	24.59	19.85			
	3	26.71	18.12			
	4	22.86	19.19			

Control—no environmental enrichment, Treatment—environmental enrichment by rubber sticks and Italian ryegrass hay; aggressive bout: starts after 5 consecutive aggressive counts and ends after 5 consecutive non-aggressive counts. Ear biting bout: starts after 3 consecutive ear biting counts and ends after 5 consecutive non-ear biting counts. Tail biting bout: starts after 3 consecutive tail biting counts and ends after 5 consecutive non-tail biting counts.

**Table 6:** Daily trends in agonistic behavior over the 28-day study period.

	Group		*p*-Value		
Parameter	Control (Mean)	Treatment (Mean)	Group	Day	Group × Day
Overall aggressive count per day	42.43	20.61	<0.001	<0.001	<0.001
Overall aggressive duration (s) per day	1005.21	389.96	<0.001	<0.001	<0.001
Overall aggressive duration per bout (s)	23.09	16.98	<0.001	<0.001	<0.001
Overall ear biting count per day	241.50	91.82	0.068	0.005	0.043
Overall ear biting duration (s) per day	6173.72	1862.39	<0.001	<0.001	0.003
Overall ear biting duration per bout (s)	25.18	20.52	<0.001	0.002	0.008
Overall tail biting count per day	100.82	30.86	<0.001	<0.001	<0.001
Overall tail biting duration (s) per day	2556.41	590.33	<0.001	<0.001	<0.001
Overall tail biting duration per bout (s)	24.69	19.10	<0.001	0.189	0.042

Control—no environmental enrichment, Treatment—environmental enrichment by rubber sticks and Italian ryegrass hay; aggressive bout: starts after 5 consecutive aggressive counts and ends after 5 consecutive non-aggressive counts. Ear biting bout: starts after 3 consecutive ear biting counts and ends after 5 consecutive non-ear biting counts. Tail biting bout: starts after 3 consecutive tail biting counts and ends after 5 consecutive non-tail biting counts. Control and Treatment values represent overall descriptive means across the 28-day observation period; *p*-values were obtained from linear mixed models with Group, Day (linear), and Group × Day as fixed effects.

**Table 7:** Ear and tail biting score of pig.

Parameter	Control (Mean ± SEM)	Treatment (Mean ± SEM)	*p*-Value
Ear biting score	1.11 ^a^ ± 0.05	0.38 ^b^ ± 0.07	<0.001
Tail biting score	0.88 ^a^ ± 0.05	0.35 ^b^ ± 0.08	0.001

Control—no environmental enrichment, Treatment—environmental enrichment by rubber sticks and Italian ryegrass hay; means with different superscripts are significantly different (*p* < 0.05).

**Table 8:** Blood biochemical parameters of the pig.

Parameter	Control (Mean ± SEM)	Treatment (Mean ± SEM)	*p*-Value
Glucose (mg/dL)	104.33 ± 2.59	106.83 ± 2.99	0.534
Creatine kinase (U/L)	1055.13 ± 263.79	973.17 ± 194.99	0.811
Lactate dehydrogenase (U/L)	591.11 ± 60.18	474.55 ± 34.70	0.096
Free fatty acid (µEq/L)	85.42 ^a^ ± 6.05	65.29 ^b^ ± 3.49	0.028
Cortisol (µg/dL)	2.14 ± 0.31	1.56 ± 0.19	0.127

Control—no environmental enrichment, Treatment—environmental enrichment by rubber sticks and Italian ryegrass hay; means with different superscripts are significantly different (*p* < 0.05).

**Table 9:** Fecal score of pig.

		Group		*p*-Value		
Parameter	Week	Control (Mean)	Treatment (Mean)	Group	Week	Group × Week
Fecal score	0 *	1.25	1.50	0.004	<0.001	0.006
	1	0.38	0.31			
	2	0.38	0.06			
	3	0.38	0			
	4	0.38	0			

Control—no environmental enrichment, Treatment—environmental enrichment by rubber sticks and Italian ryegrass hay, 0 *—initial day of the study.

## Data Availability

Data presented in this study are available upon request from the corresponding author. The trained YOLOv8 model weights, annotation schema (ethogram and labeling guidelines), and the rule-based bout-detection algorithm are available from the corresponding author upon reasonable request. A schematic description of the bout-identification algorithm is provided in Supplementary Algorithm S1. Representative labeled images and short evaluation video clips used for model validation can be shared for academic, non-commercial purposes, subject to ethical approval and institutional data-sharing regulations. Raw continuous video recordings are not publicly available due to animal welfare, privacy, and institutional restrictions.

## Supplementary Materials

The following supporting information can be downloaded at: https://www.mdpi.com/article/10.3390/biology15030255/s1. Video S1: Prediction of aggressive and ear biting behavior in the recorded video, Video S2: Prediction of tail biting behavior in the recorded video. Supplementary Algorithm S1 for rule-based identification of agonistic behavior bouts.
