# Supplementary material for: Environmental Enrichment and Agonistic Behavior in Post-Weaning Pigs: A Pilot Study Using Artificial Intelligence
Source: Biology (Basel). 2026 Jan 30;15(3):255. doi: 10.3390/biology15030255 (PMC12896451; doi:10.3390/biology15030255)
Supplement: Supplementary file 1 [file biology-15-00255-s001.zip › biology-4069200-supplementary/Supplementary Algorithm S1.pdf]

Supplementary Algorithm S1

Rule-based identification of agonistic behavior bouts

Input:

- Frame-level detections at 1 frame per second
- Behavior classes: aggressive, ear biting, tail biting
- Temporal thresholds defined in Table 2

Algorithm:

1. For each video and each behavior class independently:
2. Initialize `bout_active = False`
3. Scan detections sequentially at 1-second resolution
4. If a behavior is detected continuously for:
    - ≥5 consecutive seconds (aggressive), or
    - ≥3 consecutive seconds (ear biting or tail biting), then mark the **start of a bout**
5. While `bout_active = True`:
    - Continue accumulating bout duration for each second the behavior is detected
6. If the behavior is absent for ≥5 consecutive seconds:
    - Mark the **end of the bout**
    - Record bout start time, end time, and duration
    - Set `bout_active = False`
7. Discard detections that do not meet the minimum bout duration criteria
8. Allow simultaneous bouts of different behavior classes to occur independently
9. Repeat until the end of the video

Output:

- Total number of bouts per day
- Total bout duration per day (seconds)
- Mean duration per bout (seconds)
